# Resistance of HEK-293 and COS-7 cell lines to oxidative stress as a model of metabolic response

**DOI:** 10.3389/abp.2025.14164

**Published:** 2025-06-12

**Authors:** Monika Sapeta-Nowińska, Katarzyna Sołtys, Katarzyna Gębczak, Ewa Barg, Piotr Młynarz

**Affiliations:** ^1^ Department of Biochemistry, Molecular Biology and Biotechnology, Faculty of Chemistry, Wrocław University of Science and Technology, Wrocław, Poland; ^2^ Department of Basic Medical Science, Wrocław Medical University, Wrocław, Poland

**Keywords:** oxidative stress, metabolomics, reactive oxygen species (ROS), cell culture, TCA cycle

## Abstract

Oxidative stress (OS), arising from an imbalance between reactive oxygen species (ROS) production and antioxidant defenses, plays a pivotal role in cellular dysfunction and the pathogenesis of numerous diseases. This study evaluates the impact of oxidative stress induced by hydrogen peroxide on the metabolomic profiles of the human embryonic kidney (HEK-293) and African green monkey kidney (COS-7) cell lines. Viability (MTT) and free radical accumulation (DCF-DA) assays confirmed a dose-dependent cytotoxic effect of hydrogen peroxide, with COS-7 cells exhibiting greater resistance and producing lower levels of intracellular ROS compared to HEK-293. Metabolomic profiling was conducted using nuclear magnetic resonance spectroscopy (^1^H NMR) to identify and quantify metabolic changes. Exposure to a free radical inducer significantly altered both intracellular and extracellular metabolites compared to control H_2_O_2_-free samples. The analysis revealed common changes in intracellular metabolites between the two lines, including glutamate, NAD^+^, glutathione, ATP/ADP, AMP, and pyruvate — key molecule for mitochondrial function, as well as extracellular metabolites such as glutamate, glutamine, acetate, lactate, and pyruvate. Metabolomic differences observed in COS-7 cells suggest a potentially greater capacity for metabolic adaptation to oxidative stress. These included elevated levels of branched-chain amino acids (BCAA), supporting energy production, and increased formate production, which may aid purine synthesis and cellular resilience. These findings highlight the distinct metabolic adaptations of COS-7 cells to oxidative stress in comparison to the HEK-293 cell line. They also provide insights into the direct cellular responses to altered redox potential, offering possible therapeutic strategies aimed at targeting metabolic pathways to mitigate oxidative stress.

## Introduction

Oxidative stress (OS), first described by Helmut Sies in 1985, is a critical factor in the development of numerous diseases (Sies, 2015; Teleanu et al., 2022; Zheng et al., 2022). It arises from an imbalance between reactive oxygen species (ROS) production and antioxidant defenses. ROS, primarily generated in the mitochondrial respiratory chain, are by-products of normal cellular metabolism. However, environmental factors such as pollution, microbial infections, radiation, and smoking can exacerbate ROS production, increasing the risk of cellular damage (Valko et al., 2007; Aseervatham et al., 2013). To counteract ROS-induced harm, cells rely on antioxidant enzymes such as superoxide dismutase (SOD), catalase (CAT), and glutathione peroxidase (GPX) (Nathan and Cunningham-Bussel, 2013).

**FIGURE 1:**
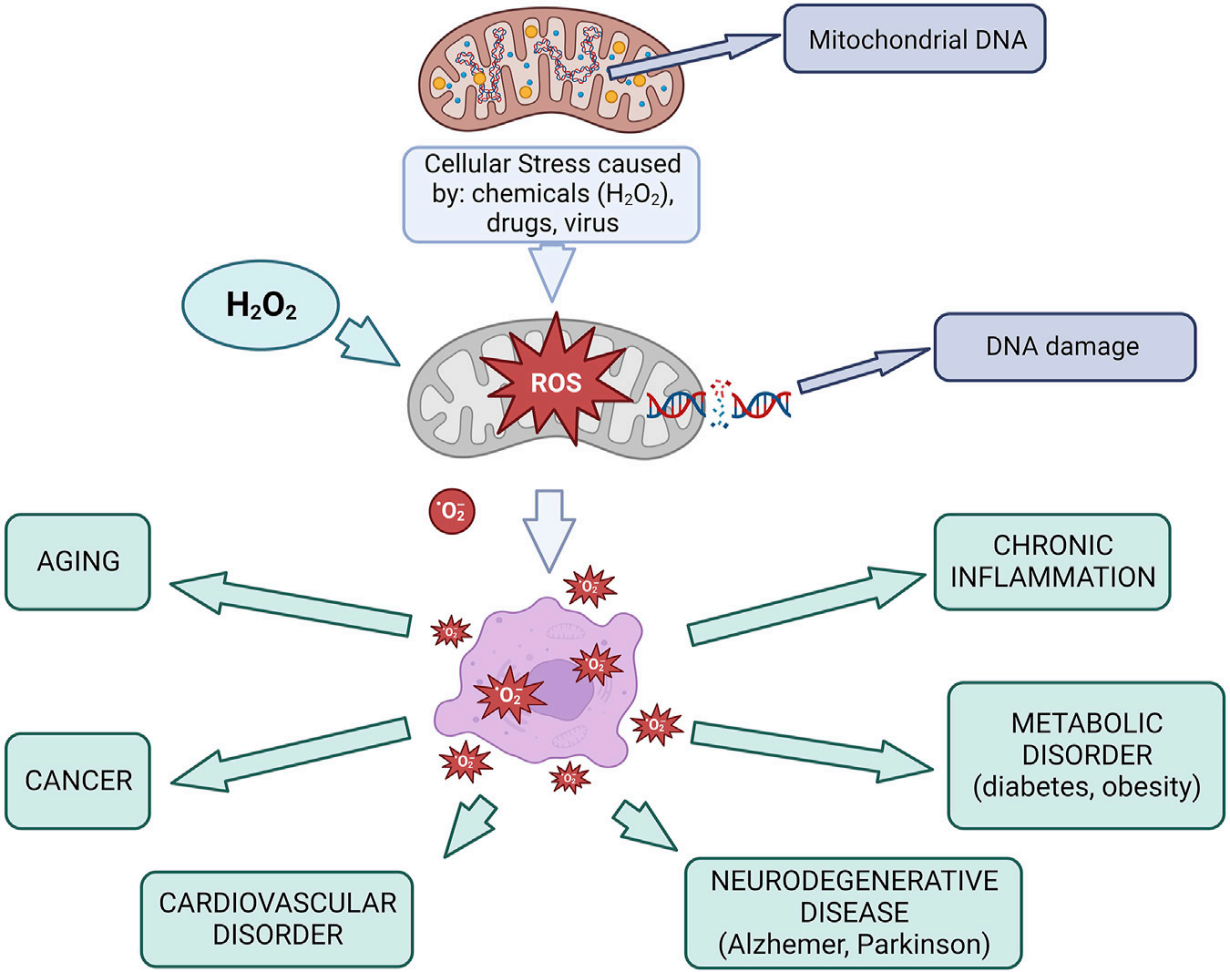
Mitochondria in oxidative stress and diseases Created using BioRender.com.

While physiological ROS levels support processes like immune defense and wound healing, excessive ROS can overwhelm antioxidant defenses, triggering oxidative stress (Ďuračková, 2010; Bhattacharyya et al., 2014). This leads to cellular damage and inflammation, which contribute to diseases such as cardiovascular disorders (e.g., coronary artery disease, hypertension, and atherosclerosis), vascular diseases (e.g., rheumatoid arthritis and inflammatory bowel disease), neurodegenerative conditions (e.g., Alzheimer’s and Parkinson’s diseases), respiratory disorders (e.g., asthma, acute lung injury, and cystic fibrosis), and cancer (Madamanchi et al., 2005; Matés et al., 2012; Bezerra et al., 2023; Olufunmilayo et al., 2023) (Figure 1).

Hydrogen peroxide (H_2_O_2_) is a widely used agent to experimentally induce oxidative damage in cell models due to its ability to generate hydroxyl radicals (⋅OH) via the Fenton reaction. It may also directly oxidize thiol groups in redox-sensitive proteins, leading to functional modifications. High H_2_O_2_ concentrations (>50 µM) are cytotoxic to most cell types, causing DNA strand breaks, nitrogen base alterations, and DNA-protein cross-links (Halliwell, Clement, and Long, 2000; Kołodziej et al., 2018; Ransy et al., 2020). Elevated ROS levels also lead to mitochondrial DNA damage, chromosomal aberrations, and mutations, which promote the development of oxidative stress-related diseases (Singh et al., 2019).

To study the effects of oxidative stress, various assays are employed. For example, 2′,7′-dichlorodihydrofluorescein diacetate (DCF-DA) measures ROS levels, while SOD, CAT, and GPX assays assess antioxidant activity (Weydert and Cullen, 2010). Additionally, the MTT (3-(4,5-dimethylthiazol-2-yl)-2,5-diphenyltetrazolium bromide) assay evaluates cell viability by measuring mitochondrial activity. Metabolomics, a comprehensive tool for analyzing biochemical changes, is particularly effective in investigating oxidative stress-induced metabolic alterations (Liu et al., 2011; Deidda et al., 2018).

Studies on OS are typically conducted using *in vitro* models, where cell cultures provide a platform to investigate oxidative stress, cellular toxicity, and adaptive responses. These models also help identify compounds with therapeutic potential (Gille and Joenje, 1992; Chung et al., 2022). Cells exposed to pro-oxidant agents like menadione, potassium bromate, or hydrogen peroxide (H_2_O_2_) represent a gold standard model to examine diverse cellular responses, leading to various cellular reactions and pathways in response to oxidative stress induction (Goffart et al., 2021). This study examines the effects of H_2_O_2_-induced oxidative stress on human embryonic kidney (HEK-293) and African green monkey kidney (COS-7) cells. The kidney cells, being rich in mitochondria, are highly susceptible to oxidative stress, a key factor in kidney diseases—a growing global health concern (Daenen et al., 2019; Gyurászová et al., 2020). The primary aim is to identify specific metabolic changes linked to ROS-induced mitochondrial dysfunction, particularly those involving the tricarboxylic acid (TCA) cycle. By comparing the resilience of these two cell lines, this work provides novel insights into the biochemical alterations and adaptive responses to oxidative stress at the cellular level.

## Material and methods

### Cell culture

The HEK-293 (human embryonic kidney cells; ECACC cat. no. 85120602) and COS-7 (fibroblast-like cell line derived from African green monkey kidney tissue; ECACC cat. no. 87021302) cell lines were cultured in Dulbecco’s Modified Eagle’s Medium (DMEM, Sigma Aldrich, UK) supplemented with 10% fetal bovine serum (FBS, Biowest S181H-500) and 100 μg/mL penicillin–100 μg/mL streptomycin, in a humidified incubator at 37°C with 5% CO_2_ and 95% humidity. The cells were maintained in 75 cm^2^ or 150 cm^2^ flasks, with the medium replaced twice a week. The cells were passaged twice to three times a week, and the culture was split once confluence reached 85%–90%.

### Oxidative stress induction

Oxidative stress was induced by treating cells with hydrogen peroxide (H_2_O_2_, 30% H_2_O_2_, Sigma, EMSURE, cat. no. 1072090250) at various concentrations (100 nM, 1 μM, 0.1 mM, 1 mM, 3.19 mM, 6.3 mM, 12.3 mM, and 37.8 mM) for 30 min (PBS buffer diluent; Sigma Aldrich, UK, P2272). For each experiment, cells were pre-incubated in 96-well plates (2.1 × 10^4^ cells/well for HEK-293, and 3.2 ×10^4^ cells/well for COS-7) for 24 h to allow cell adhesion (5% CO_2_ and 95% humidity at 37°C). After the incubation, H_2_O_2_ was removed, and the cells were rinsed with phosphate-buffered saline (PBS) before subsequent assays.

### Cell viability assay - MTT assay

Cells viability was determined using the MTT (3-(4,5-dimethylthiazol-2-yl)-2,5-diphenyltetrazolium bromide) colorimetric assay. After oxidative stress induction, HEK-293 and COS-7 cell lines were incubated with MTT solution (BioReagent, >97.5%, Sigma Aldrich, UK) for 2 h.The medium was removed and formazan crystals were dissolved in isopropanol (99.7%, PolAura, Poland) for 30 min in the dark. Absorbance was measured at 570 nm using a Victor2 microplate reader (Perkin Elmer). Cell viability was calculated as the ratio of the absorbance of treated samples to that of untreated control samples (control absorbance), expressed as a percentage. Cell viability was calculated as follows:

$$Cells\;viability=\frac{sample\;absorbance}{control\;absorbance}\;\times 100\%$$

### Measurement of reactive oxygen species production - DCF-DA assay

Reactive oxygen species (ROS) production was assessed using the DCF-DA (2′,7′-dichlorodihydrofluorescein diacetate) assay. The DCF-DA (Sigma Aldrich, UK) solution was freshly prepared by dissolving DCF-DA in 100% ethanol and diluting it to a working concentration of 10 µM in the medium. After H_2_O_2_ treatment, two cell lines were incubated with the DCF-DA solution for 1 h under dark conditions. Fluorescence intensity, which correlates with intracellular ROS levels, was measured using a Victor2 microplate reader (excitation wavelength: 485 nm; emission wavelength: 530 nm). Results were expressed as fluorescence ratios (E/E_0_), where E represents the fluorescence intensity of the treated sample and E_0_ represents the control.

### Sample preparation and ^1^H NMR measurement

For metabolomic analysis, cells were cultured in 75 cm^2^ flasks (HEK-293) and 150 cm^2^ flasks (COS-7) until reaching confluence (3.0 × 10^7^ HEK-293 cells or 8.0 × 10^6^ COS-7 cells). The difference in flask size and final cell numbers reflects the distinct morphology and growth characteristics of the two cell lines. HEK-293 cells proliferate more rapidly and are smaller in size, while COS-7 cells are larger and require greater surface area to reach comparable confluence. Cell harvesting was performed at ∼90% confluence for both lines to ensure consistency in metabolic state. Following treatment with H_2_O_2_ at the selected concentrations (100 nM, 1 μM, 0.1 mM, 3.19 mM, and 37.8 mM), the culture medium containing extracellular metabolites was carefully collected and stored on ice for further analysis. The cells were then rinsed with PBS to remove any residual medium. After that, the cells were placed on ice, and 3 mL of cold methanol: water (3:1) was added twice. The samples with cells and methanol were stored at −80°C for further analysis.

For metabolomic analysis, six repeats of extracellular metabolites from each concentration and three repetitions of intracellular metabolites were performed. The samples containing cells and methanol were homogenized using a metal ball. The homogenization process was carried out twice for 5 min at 50 Hz, with a freezing period between each session. The homogenized samples were then centrifuged at 4°C at 14.000 RPM for 10 min. After centrifugation, the supernatant was collected and evaporated using a Speedvac at 500 RPM at 45°C. Solutions containing extracellular metabolites were not homogenized. Following evaporation, 600 µL of cell buffer (1.107 g NaH_2_PO_4_, 0.263 g Na_2_HPO_4_, 0.005g TSP, 50 mL D_2_O, 50 mL H_2_O) was added to the precipitated samples. A final volume of 550 µL was transferred to NMR tubes.

^1^H NMR spectra were acquired at an operating frequency of 600.58 MHz on a BRUKER ULTRASHIELD™ PLUS AV2 spectrometer. The PULPROG sequence, cpmgpr1d, was used for analysis. The processing parameters were as follows: relaxation delay (RD) = 3.5 s, acquisition time = 2.75 s, total number of scans = 128, mixing time = 125 ms, and time domain data points = 64 K. Spectra were manually phased using TopSpin 3.2 software (Bruker, GmbH, Germany). Baseline correction was performed using the Whittaker smoother algorithm in MestReNova software (MestReNova v. 11.0.3, Qingdao, China), and the spectra were referenced to the TSP resonance at 0.0 ppm.

### Data analysis and statistical methods

All ^1^H NMR spectra were exported to MATLAB (R2014a, Natick, MA, United States) for preprocessing. The water region (4.65–5.22 ppm) was excluded from the analysis. The spectra were corrected for baseline and phase distortions, and referenced to the trimethylsilyl propanoic acid (TSP) signal at δ 0.00 to assess changes in endogenous metabolites related to hydrogen peroxide toxicity. Resonance signal alignment was performed using the correlation-optimized warping (COW) and interval correlation shifting (icoshift) algorithms.

All spectra were normalized using Probabilistic Quotient Normalization (PQN), applied separately within each cell line group to account for differences in baseline signal intensity and cell number. For statistical analysis, data from 22 extracellular metabolites (HEK-293), 20 extracellular metabolites (COS-7), and 21 intracellular metabolites (HEK-293, COS-7) were considered. Metabolites were identified using Chenomx software (v 8.4, Chenomx Inc., Edmonton, Canada) and verified through the Human Metabolome Database^1^ and published literature. For each metabolite, only one representative signal was used in the analysis, with NMR assignments detailed in Supplementary Tables S1, S2. Univariate analysis was conducted using MATLAB, with the normality of data distributions assessed via the Shapiro-Wilk test. Depending on the normality results, statistical comparisons were performed using either Student’s t-test (for parametric data) or the Mann-Whitney U test (for non-parametric data). A p-value ≤0.05 was considered statistically significant.

### Multivariate data analysis

For multivariate analysis, extracellular metabolites from HEK-293 (22 metabolites) and COS-7 (20 metabolites), as well as intracellular metabolites (HEK-293 and COS-7, 21 metabolites each), were analyzed. The data matrix was created from metabolite concentrations for each sample and transformed in SIMCA-P software (v 17.0, Umetrics, Umeå, Sweden). Data were scaled using UV scaling before chemometric analysis. Principal Component Analysis (PCA) was applied to the data, and results with p ≤ 0.05 were considered statistically significant.

### Statistical analysis of the result of biological activity assay

Statistical analysis of the biological activity assays was performed using the Dixon method for outlier detection. Results were assessed for statistical significance using Student's t-test (for normally distributed data) or Mann-Whitney U test (for non-normally distributed data). A p-value ≤0.05 was considered significant. The analysis was conducted using STATISTICA 13.3.

## Results

### Biological activity tests

#### Cytotoxicity of hydrogen peroxide in HEK-293 cells, and COS-7 cells

**FIGURE 2:**
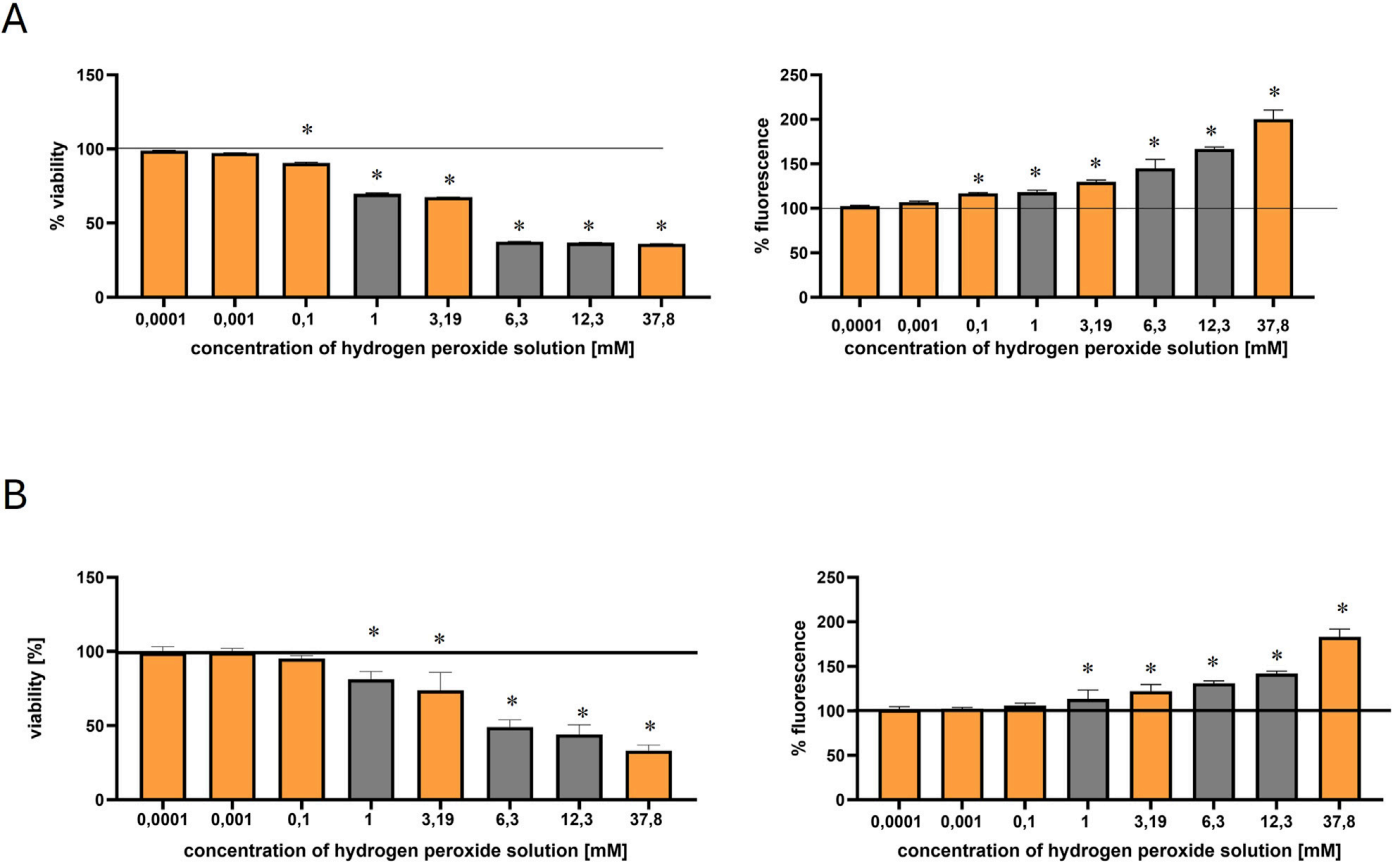
Effect of hydrogen peroxide (H_2_O_2_) concentration on HEK-293 and COS-7 cell viability and intracellular ROS levels. Orange bars indicate the H_2_O_2_ concentrations selected for metabolomic profiling. The black solid line represents the control, normalized to 100% viability or fluorescence. Statistically significant differences (p ≤ 0.05) are marked with an asterisk (*). **(A)** Viability of HEK-293 cells (left) measured by the MTT assay and intracellular ROS levels (right) measured by the DCF-DA assay after 30-min incubation with increasing concentrations of H_2_O_2_. **(B)** Viability of COS-7 cells (left) and ROS levels (right) assessed under the same experimental conditions.

The cytotoxic effects of hydrogen peroxide were evaluated in both HEK-293 and COS-7 cell lines using the MTT and DCF-DA assays (Figures 2A,B). The range of tested concentrations included 100 nM, 1 μM, 0.1 mM, 1 mM, 3.19 mM, 6.3 mM, 12.3 mM, and 37.8 mM. The orange bars indicate the concentrations selected for metabolomic profiling: 100 nM, 1 μM, 0.1 mM, 3.19 mM, and 37.8 mM.

In HEK-293 cells, viability began to decline at concentrations ≥0.1 mM, indicating the onset of oxidative stress. According to Bian et al. (2015), an IC_50_ of approximately 0.96 mM (after 2 h of exposure) confirmed the susceptibility of this cell line to H_2_O_2_ induced damage, supporting its use as a model for studying early oxidative stress responses (Bian et al., 2015).

In contrast, the COS-7 cell line exhibited lower sensitivity, for instance, incubation of COS-7 cells with H_2_O_2_ at 200 μM for 24 h resulted in 83.2% viability, while at 400 μM, at the same time viability decreased to 67.2% (Yoon et al., 2016).

In the present study, both cell lines exhibited a dose-dependent decrease in viability and a corresponding increase in intracellular ROS production (measured by DCF-DA fluorescence). However, COS-7 cells maintained higher viability compared to HEK-293 (Figures 2A,B). At 0.1 mM H_2_O_2_, HEK-293 cells showed a more pronounced reduction in viability (∼89%) compared to COS-7 (∼95%).

#### Reactive oxygen species induced by hydrogen peroxide

The generation of reactive oxygen species (ROS) in HEK-293 and COS-7 cells following 30-min incubation with hydrogen peroxide was assessed using the DCF-DA fluorescence assay (Figures 2A,B). As shown in Figure 2, the lowest tested concentrations (100 nM and 1 μM) did not induce a significant increase in ROS levels in either cell line, when compared to the control. However, exposure to 0.1 mM H_2_O_2_ in HEK-293 cells resulted in a statistically significant rise in fluorescence intensity, indicating increased intracellular ROS production. In contrast, COS-7 cells showed a more gradual increase in ROS levels, with statistically significant changes observed at higher concentrations. Thus, this concentration led to a modest increase in ROS levels in both lines, with fluorescence rising to ∼116% in HEK-293 and ∼103% in COS-7 (Figures 2A,B).

### Metabolomics analysis

#### Analysis of intracellular metabolism for HEK-293 and COS-7 cell lines

**FIGURE 3:**
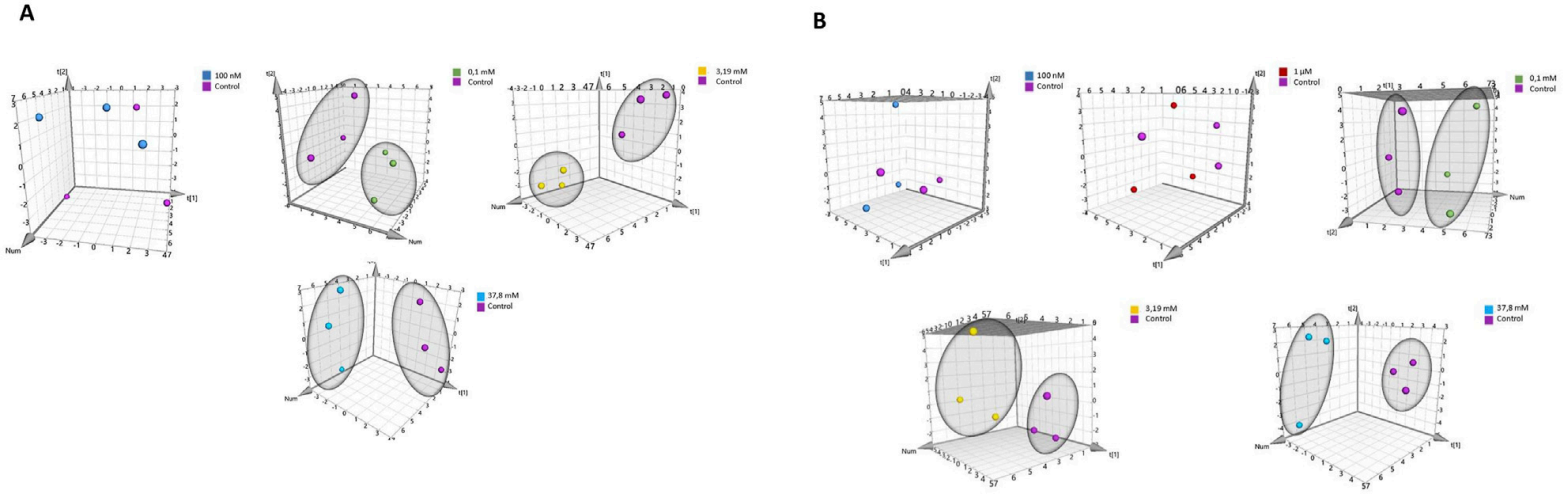
PCA score plot of ^1^H NMR data obtained from comparison of groups controls with: 100 nM, 1 μM, 0.1 mM, 3.19 mM, and 37.8 mM for cell lines. **(A)** for HEK-293 cell line. **(B)** for COS-7 cell line.

Representative signals for each metabolite in both the control and 3.19 mM hydrogen peroxide-treated samples for HEK-293 and COS-7 cell lines, along with their ^1^H NMR spectra, are presented in the Supplementary Material (Supplementary Table S1; Supplementary Figures S1A,B *)*. A total of 23 intracellular metabolites were identified. Due to the overlapping of some signals from some intracellular metabolites - creatine phosphate/creatine at 3.02 ppm, and AXP (ATP/ADP/AMP) at 8.26 ppm, were not included in the statistical and multivariate analyses Supplementary Figure S1. Additionally, the signal originating from methanol (used as a solvent for extraction) was also excluded from the analyses. Multivariate data analysis was performed to identify patterns and trends among the compared samples. The analysis utilized Principal Component Analysis (PCA), a classic unsupervised method were employed for: projection, visualization, and trend identification, as well as the detection of outliers in trials. The analysis results are depicted in Figures 3A,B. Supplementary Tables S3, S4 outline the parameters of the PCA models for HEK-293 and COS-7 cell lines, demonstrating highly effective sample clustering.

**FIGURE 4:**
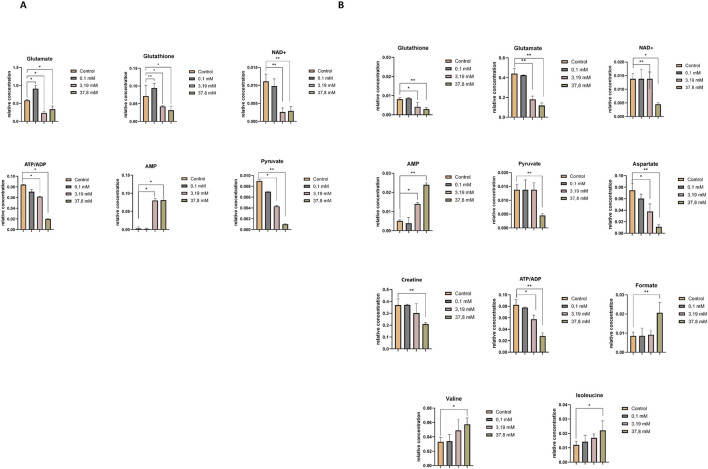
Relative concentrations of intracellular metabolites showing statistically significant differences between the control and treatment groups exposed to increasing concentrations of hydrogen peroxide (0.1 mM, 3.19 mM, and 37.8 mM). **(A)** Statistically significant changes in metabolite levels in the HEK-293 cell line. **(B)** Statistically significant changes in metabolite levels in the COS-7 cell line. Significant differences are indicated as follows: *p < 0.05, **p < 0.01, ***p < 0.001. Note: “ATP/ADP” represents a partially overlapping NMR signal region that includes both ATP and ADP resonances, which cannot be unambiguously separated due to limited spectral resolution.

Supplementary Tables S5, S6 compare the relative concentrations of metabolites in controls and samples incubated at different concentrations of hydrogen peroxide. The metabolomic differences induced by the highest concentration of hydrogen peroxide and the concentration triggering oxidative stress in HEK-293 and COS-7 cell lines are illustrated in Figures 4A,B. Common changes in metabolites were observed in both investigated cell lines with increasing relative concentrations of adenosine monophosphate (AMP) and decreasing concentrations of adenosine-5′-triphosphate/adenosine-5′-diphosphate (ATP/ADP – overlapped signals), nicotinamide adenine dinucleotide oxidized (NAD^+^), glutamate, glutathione, and pyruvate.

Additionally, in the COS-7 cell line, compared to the control without H_2_O_2_, statistically significant decreases in the relative concentrations of aspartate and creatine were observed, along with an increase in formate, isoleucine, and valine (Figure 4B).

#### Analysis of extracellular metabolism for HEK-293 cell lines and COS-7 cell lines

Supplementary Figures S1, S2 show the ^1^H NMR spectra of HEK-293 cells incubated without H_2_O_2_ (control) and with 3.19 mM H_2_O_2_. Twenty metabolites were identified in the control samples (Supplementary Figure S3), while 25 were observed in H_2_O_2_-treated samples (Supplementary Figure S2). Additional signals of metabolites detected in the treated samples included unknown_1 (8.34 ppm), unknown_2 (8.27 ppm), unknown_3 (2.7 ppm), creatine phosphate/creatine (excluded from analysis) and hypoxanthine. From the spectra the same signals were excluded from analyses as for intracellular metabolites. Representative signals for each metabolite, both in control and 3.19 mM H_2_O_2_ treated cell line samples, are listed in Supplementary Table S2.

Similarly, Supplementary Figures S4, S5 illustrate the spectra of COS-7 cells incubated with and without H_2_O_2_. Control samples (Supplementary Figure S5) revealed 19 metabolites, while H_2_O_2_-treated samples (Supplementary Figure S4) showed 23 metabolites, including unknown_1 (8.34 ppm), unknown_2 (8.27 ppm), unknown_3 (2.7 ppm), and creatine phosphate/creatine (excluded from analysis). Metabolite signals for both control and treated COS-7 samples are also detailed in Supplementary Table S2.

**FIGURE 5:**
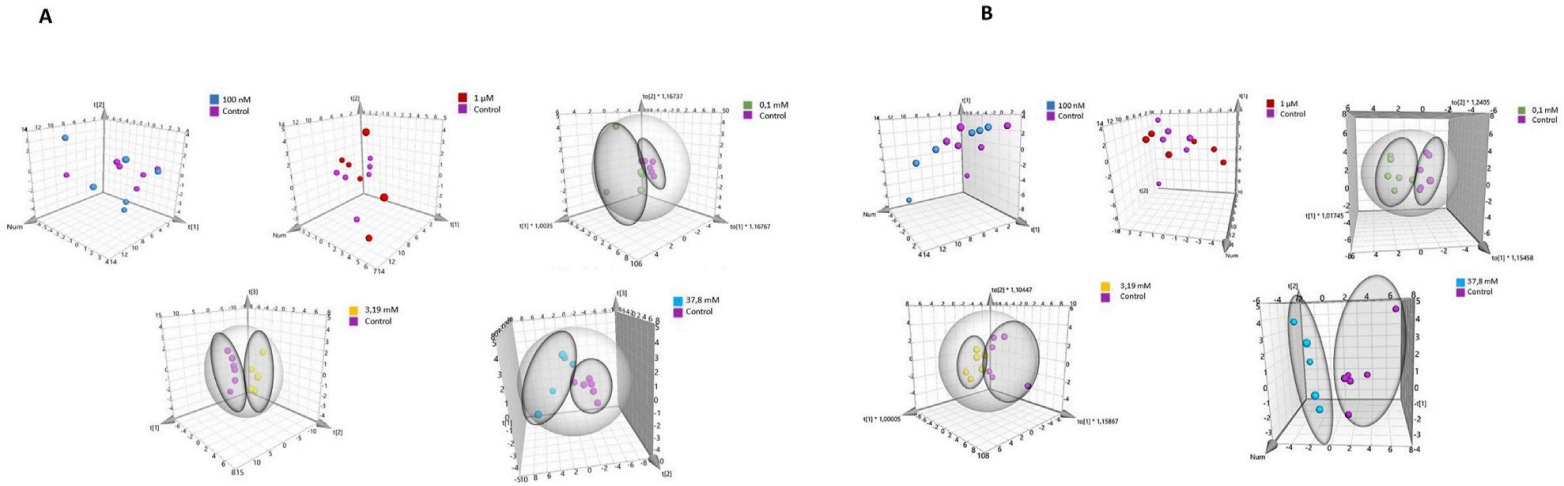
PCA score plot of ^1^H NMR data obtained from comparison of groups controls with various concentrations of H_2_O_2_ for different cell lines. **(A)** for HEK-293 cell line. **(B)** for COS-7 cell line.

Principal Component Analysis (PCA) was conducted to compare sample differentiation depending on hydrogen peroxide concentrations in both cell lines, including controls and treatments with 100 nM, 1 μM, 0.1 mM, 3.19 mM, and 37.8 mM H_2_O_2_. Results for HEK-293 and COS-7 cell lines are summarized in Supplementary Tables S7, S8. In HEK-293 cells, PCA revealed clear grouping trends (Figure 5A), particularly at concentrations ≥0.1 mM. Similar clustering patterns were observed in COS-7 cells at H_2_O_2_ concentrations of 0.1 mM, 3.19 mM, and 37.8 mM (Figure 5B), with no grouping evident between control and low-concentration treatments (100 nM and 1 μM).

**FIGURE 6:**
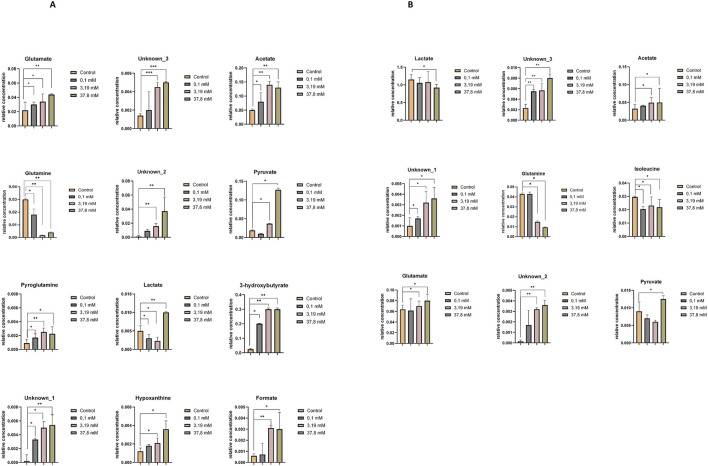
Relative concentrations of extracellular metabolites showing statistically significant differences between the control and treatment groups exposed to increasing concentrations of hydrogen peroxide (0.1 mM, 3.19 mM, and 37.8 mM). **(A)** Statistically significant changes in metabolite levels in the HEK-293 cell line. **(B)** Statistically significant changes in metabolite levels in the COS-7 cell line. Significant differences are indicated as follows: *p ≤ 0.05, **p < 0.01, ***p < 0.001.

Supplementary Table S9 (HEK-293 cell lines) and S10 (COS-7 cell line) present a comparison of the relative concentrations of metabolites in controls and samples incubated at different concentrations of H_2_O_2_. The metabolomic differences between the highest concentration of H_2_O_2_ and the induced oxidative stress concentration (0.1 mM) for HEK-293 and COS-7 cell lines are illustrated in Figures 6A,B.

Upon comparing control samples in the HEK-293 cell line with these incubated in a 0.1 mM H_2_O_2_ solution, statistically significant differences in the levels of several metabolites were identified, including glutamate, acetate, pyroglutamine, glutamine, 3-hydroxybutyrate, lactate, and unknown_1. At the highest H_2_O_2_ concentrations, additional significant changes were observed in pyruvate, unknown_3, unknown_2, formate, and hypoxanthine (Figure 6A).

In the COS-7 cell line, statistically significant alterations in metabolite levels—including isoleucine, unknown_3, and unknown_1 — were detected following incubation with a 0.1 mM H_2_O_2_ solution, compared to control samples. Additionally, significant metabolite changes were observed upon treatment with higher concentrations of H_2_O_2_ (3.19 mM and 37.8 mM) in comparison to controls. These included an increase in the relative concentrations of glutamate, acetate, unknown_2, unknown_3, and unknown_1, and a decrease in glutamine, lactate, and isoleucine (Figure 6B).

## Discussion

The biological impact of hydrogen peroxide exposure was first confirmed through MTT and DCF-DA assays, which revealed clear, concentration-dependent responses in both HEK-293 and COS-7 cell lines. A gradual reduction in cell viability was observed starting from 0.1 mM H_2_O_2_, while ROS production measured by DCF-DA fluorescence, increased significantly at the same threshold, especially in HEK-293 cells. These results indicate that even short-term exposure (30 min) to moderate concentrations of hydrogen peroxide is sufficient to trigger oxidative stress. Notably, COS-7 cells retained higher viability and exhibited a more moderate increase in ROS levels, suggesting greater oxidative resilience. These findings provided a basis for the metabolomic analysis, shedding light on how cells adapt to oxidative stress and which processes are most affected. Concentrations of 100 nM and 1 μM H_2_O_2_ did not elicit notable changes in the metabolite profiles of HEK-293 and COS-7 cells. However, significant metabolic shifts were detected starting at 0.1 mM hydrogen peroxide. The relatively short incubation time was chosen to capture the early-phase metabolic response to oxidative stress, consistent with previous reports indicating that exposure to a single, concentrated dose of H_2_O_2_ is sufficient to induce a measurable oxidative stress response (Gille and Joenje, 1992; Wiese et al., 1995; Deferme et al., 2013).

Both cell lines at a higher concentration of H_2_O_2_ demonstrated a decrease in key intracellular metabolites critical for energy metabolism, including glutathione (GSH), glutamate, NAD^+^, ATP/ADP, and pyruvate, alongside an increase in AMP levels. Under physiological conditions, NAD^+^ is involved in pyruvate conversion to acetyl-CoA and the electron transport chain (ETC.) function via NADH generation. Oxidative stress may disrupt this process by impairing key TCA cycle enzymes, such as isocitrate dehydrogenase and α-ketoglutarate dehydrogenase (α-KGDH), thereby reducing NADH production and ATP turnover (Tretter and Adam-Vizi, 2000; Gray, Tompkins, and Taylor, 2014). The observed decrease in pyruvate suggests disruption of glycolysis and possible oxidative damage to cells (Gray, Tompkins, and Taylor, 2014). Additionally, during oxidative stress, an increase in AMP was observed in both cell lines. Increased intracellular AMP levels indicate an energy-deprived state (Choi et al., 2001; Hawley et al., 2010; Auciello et al., 2014).

Focusing on the HEK-293 cell line, oxidative stress induced profound intracellular metabolic collapse. An increased intracellular GSH at 0.1 mM H_2_O_2_ indicates an early defense mechanism against OS. However, at higher concentrations, GSH depletion occurred, evidenced by elevated extracellular pyroglutamine levels, a marker of oxidative stress and GSH depletion (Gamarra et al., 2019). The crucial role of glutathione tripeptide (GSH) in protecting cells from oxidative stress is evident, as it acts as a free radical scavenger (Townsend et al., 2003). Glutamate, synthesized from glutamine, plays a vital role in GSH biosynthesis and mitochondrial function (Sappington et al., 2016). Therefore, OS-induced reductions in glutamate levels likely reflect mitochondrial impairment (Straadt et al., 2010).

In COS-7 cells increases in formate, isoleucine, and valine levels were observed, accompanied by a decrease in creatine and aspartate concentration. These changes suggest metabolic reprogramming to support energy production and mitochondrial resilience. Increased levels of branched-chain amino acids (BCAAs), such as isoleucine and valine, which enter the TCA cycle via intermediates like propionyl-CoA, acetyl-CoA, and succinyl-CoA, may reflect adaptations to maintain ATP production under oxidative conditions (Bhagavan and Ha, 2015; Wu et al., 2022). These changes are consistent with activation of mTOR signaling pathways in response to oxidative stress (Bonvini et al., 2018). Reduced aspartate levels may be also explained by mitochondrial impairment and disturbances in the TCA cycle or associated biosynthetic routes (Straadt et al., 2010). A decreasing trend in aspartate was also noted in HEK-293 cells, approaching statistical significance. Elevated formate in COS-7 cells may support nucleotide biosynthesis and oxidative stress mitigation through effects on tetrahydrofolate metabolism, although the exact mechanism remains to be clarified (Oizel et al., 2020; Pietzke et al., 2020).

Significant alterations were also observed in extracellular metabolites, including pyruvate, acetate, glutamine, glutamate, lactate, and unidentified compounds (unknown_1–3). Both cell lines exhibited elevated extracellular acetate levels, which could indicate enhanced *de novo* synthesis aimed at preserving intracellular acetyl-CoA pool during oxidative stress (Bose, Ramesh, and Locasale, 2019). As a key intermediate in central metabolism, acetate contributes to acetyl-CoA generation and provides an alternative carbon source under nutrient-limited conditions (Liu et al., 2018). A shared characteristic was the elevated extracellular pyruvate concentration observed in both COS-7 and HEK-293 cell lines, with a statistically significant increase detected in COS-7 cells at 37.8 mM H_2_O_2_ compared to HEK-293 cells at 3.19 mM. Increased extracellular pyruvate is more likely indicative of intracellular efflux due to membrane damage. Additionally, another extracellular metabolite of statistical significance is glutamine, which exhibited a decrease in extracellular medium concentration in both cell lines. It’s worth noting that glutamine serves as a precursor for glutamate, a key component in the biosynthesis of glutathione (Matés et al., 2002). As intracellular glutamine levels decrease, there is a corresponding increase in extracellular glutamate, which might result in a lower concentration of intracellular GSH. Furthermore, extracellular lactate levels showed statistically significant changes in both COS-7 and HEK-293 cell lines compared to the control. In HEK-293 cells, a decrease in lactate was observed at most concentrations, except for the highest H_2_O_2_ concentration (37.8 mM), where an increase occurred. In COS-7 cells, a statistically significant decrease in extracellular lactate was noted only at 37.8 mM H_2_O_2_.

HEK-293 cells displayed additional significant changes, including increased extracellular pyroglutamine, hypoxanthine, 3-hydroxybutyrate (BHB), and formate. Formate and hypoxanthine accumulation may reflect mitochondrial dysfunction and chemical damage caused by oxidative stress, potentially resulting in membrane rupture and the release of intracellular contents. Hypoxanthine is routinely evaluated as a marker for OS due to its association with mitochondrial dysfunctions (Makowski, 2021). Elevated BHB suggests an antioxidant response aimed at mitigating oxidative damage and supporting cellular protection (Haces et al., 2008; Rojas-Morales et al., 2020).

In general, the HEK-293 cell line displayed greater vulnerability to oxidative stress. As mentioned earlier, Bian et al. (2015) observed significant oxidative damage in HEK-293 cells, with an IC_50_ of approximately 0.96 mM following a 2-h exposure to H_2_O_2_. This study confirms the high sensitivity of HEK-293 cells to oxidative stress. In our study, significant metabolic changes and viability reduction were already evident at similar concentrations following a much shorter 30-min exposure, suggesting an early and rapid onset of oxidative stress responses. GSH depletion, alongside elevated extracellular pyroglutamine, hypoxanthine, and BHB, highlights the sensitivity of HEK-293 cells to oxidative damage. Moreover, HEK-293 cells showed increased extracellular formate and hypoxanthine levels, reflecting mitochondrial dysfunction and disruptions in purine metabolism caused by oxidative stress (Oizel et al., 2020).

**FIGURE 7:**
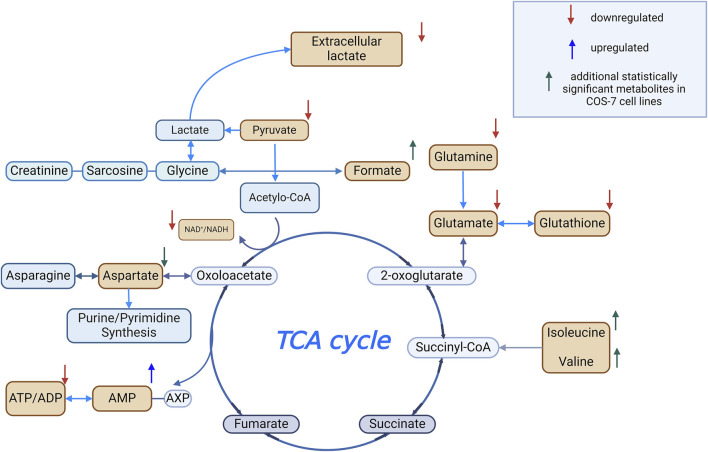
Metabolomic changes during oxidative stress Created using BioRender.com.

Our findings suggest that COS-7 cells, derived from monkey kidney tissue, may exhibit a more stable metabolic profile under oxidative stress conditions. These cells showed smaller reductions in pyruvate, elevated formate levels, and increased BCAAs, reflecting early metabolic adaptations to oxidative challenges. This observation aligns with previous studies, such as Yoon et al. (2016), which reported COS-7 cells maintaining 83.8% viability at 100 μM H_2_O_2_ and 68% at 400 μM after 24 h. Figure 7 summarizes statistically significant changes in intracellular metabolite levels observed during oxidative stress.

## Conclusion

This study demonstrates the utility of metabolomics in uncovering early cellular responses to oxidative stress induced by hydrogen peroxide (H_2_O_2_). Concentration-dependent metabolic changes were observed, including disruptions in mitochondrial function, the TCA cycle, and amino acid metabolism, accompanied by reductions in GSH and other energy-related metabolites such as ATP/ADP, and AMP – key molecules involved in cellular energy transfer. HEK-293 cells showed changes consistent with a higher susceptibility to oxidative stress, including GSH depletion, elevated oxidative stress markers, and altered purine metabolism. In contrast COS-7 cells exhibited metabolic changes that may reflect an adaptive response, including increases in branched-chain amino acids (BCAAs) and formate, which are associated with mitochondrial support and purine metabolism. These metabolic observations were in line with the results of the MTT and DCF-DA assays, which confirmed reduced viability and increased ROS levels in HEK-293 cells, and a relatively more stable redox status in COS-7 cells.

## Data Availability

The original contributions presented in the study are included in the article/Supplementary Material, further inquiries can be directed to the corresponding author.

## Footnotes

1
www.hmdb.ca
