# Supplementary material for: Resistance of HEK-293 and COS-7 cell lines to oxidative stress as a model of metabolic response
Source: Acta Biochim Pol. 2025 Jun 12;72:14164. doi: 10.3389/abp.2025.14164 (PMC12198024; doi:10.3389/abp.2025.14164)
Supplement: Supplementary file 1 [file DataSheet1.docx]

**Table S1.** Characterization of representative ^1^H NMR signals for intracellular metabolites for HEK-293, and COS-7 cell lines, were taken for quantification of relative integral used for data analysis.

| **Metabolite** | **Chemical shift (ppm)** | **Multiplicity** | **HMDB ID** |
| --- | --- | --- | --- |
| leucine | 0,956 | t | HMDB0000687 |
| isoleucine | 0,999 | d | HMDB0000172 |
| valine | 1,03 | d | HMDB0000883 |
| lactate | 1,32 | d | HMDB0000190 |
| alanine | 1,47 | d | HMDB01310 |
| acetate | 1,91 | s | HMDB0000042 |
| glutamate | 2,32 | m | HMDB0060475 |
| glutathione | 2,53 | dd | HMDB0000125 |
| pyruvate | 2,36 | s | HMDB0000243 |
| creatine | 3,92 | s | HMDB0000064 |
| O-phosphocholine | 3,21 | s | HMDB0001565 |
| glucose | 3,46 | m | HMDB0003405 |
| aspartate | 2,796 | dd | HMDB0000191 |
| myo-inositol | 4,06 | dd | HMDB0000211 |
| UDP-glucose/ UDP-N-acetyloglucosamine | 5,97 | - | - |
| NAD^+^ | 6,02 | d | HMDB0001487 |
| ATP/ADP | 6,12 | s | - |
| tyrosine | 7,18 | d | HMDB0000866 |
| phenylalanine | 7,4 | m | HMDB0000159 |
| AMP | 8,58 | s | HMDB0000045 |
| formate | 8,44 | s | HMDB0000142 |

*d-doublet, dd – doublet of doublets, s – singlet, t – triplet, m – multiplet.*

**Table S2.** Characterization of representative ^1^H NMR signals for extracellular metabolites for HEK-293, and COS-7 cell lines, were taken for quantification of relative integral used for data analysis.

| **Metabolite** | **Chemical shift (ppm)** | **Multiplicity** | **HMDB ID** |
| --- | --- | --- | --- |
| 2-hydroxyvalerate | 0,89 | t | HMDB0000531 |
| isoleucine | 0,998 | d | HMDB0000172 |
| leucine | 0,956 | t | HMDB0000687 |
| valine | 1,03 | d | HMDB0000883 |
| 3-hydroxybutyrate | 1,18 | t | HMDB0000011 |
| lactate | 1,32 | d | HMDB0000190 |
| alanine | 1,47 | d | HMDB01310 |
| acetate | 1,91 | s | HMDB0000042 |
| glutamine | 2,46 | dt | HMDB0003423 |
| glutamate | 2,32 | dt | HMDB0060475 |
| pyruvate | 2,36 | s | HMDB0000243 |
| pyroglutamate | 2,497 | m | HMDB0000267 |
| lysine | 1,72 | tt | HMDB0000182 |
| glucose | 3,46 | m | HMDB0003405 |
| threonine | 4,25 | m | HMDB0000167 |
| tyrosine | 7,18 | d | HMDB0000866 |
| phenylalanine | 7,4 | m | HMDB0000159 |
| formate | 8,44 | s | HMDB0000142 |
| unknown_3 | 2,7 | s | - |
| hypoxanthine | 8,18 | d | HMDB0000157 |
| unknown_2 | 8,27 | s | - |
| unknown_1 | 8,34 | s | - |

*d – doublet, dt – doublet of triplet, s – singlet, t – triplet, m – multiplet.*


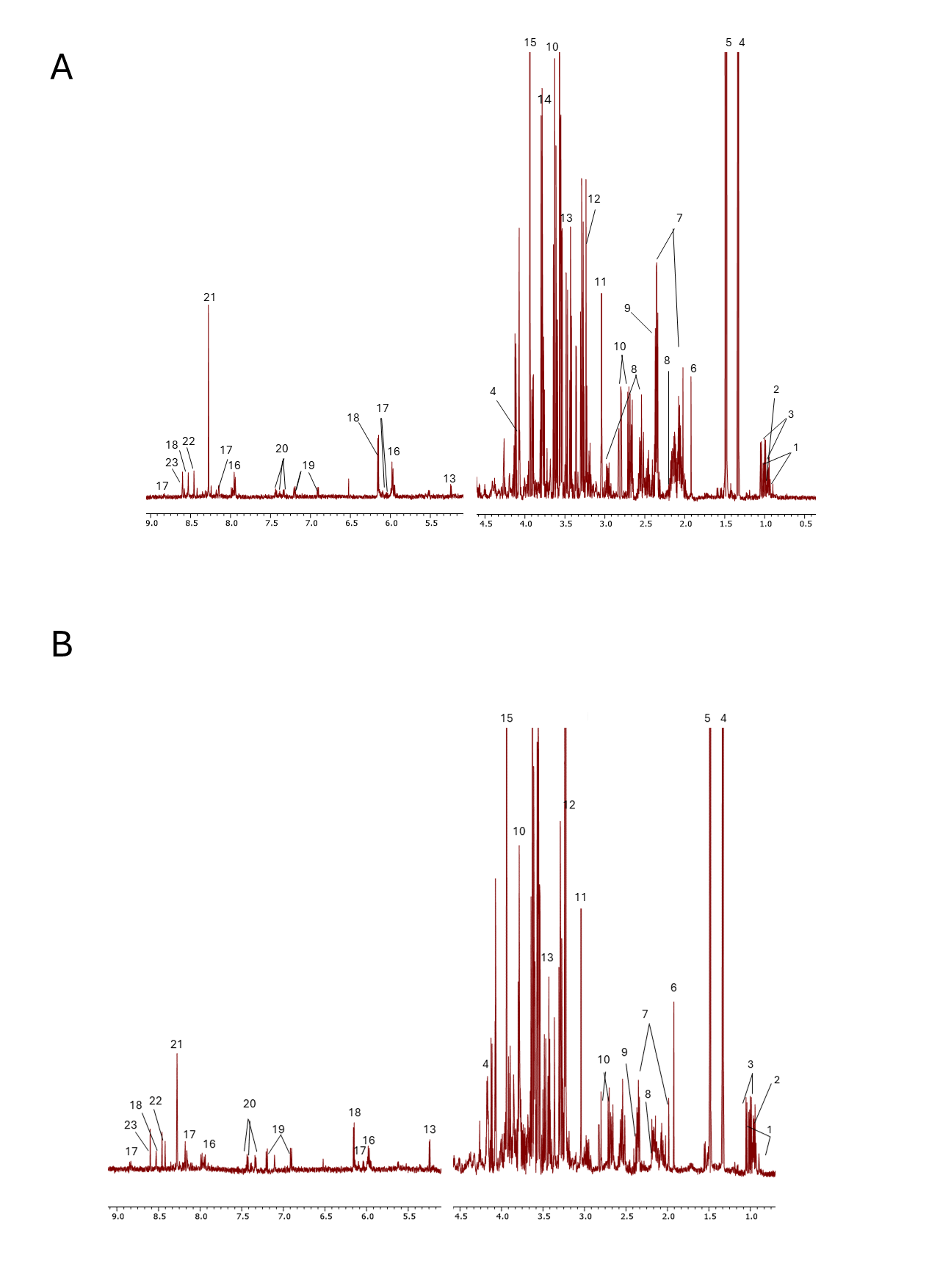
**Fig. S1. High-resolution ^1^H NMR spectrum (600 MHz) of representative spectra for cell line post-oxidative stress (3.19 mM)**

(**A)** for HEK-293 cell line**. (B)** for COS-7 cell line.

1- isoleucine; 2 - leucine; 3 - valine; 4 – lactate; 5 – alanine; 6 – acetate; 7 – glutamate; 8 – glutathione; 9-pyruvate; 10-aspartate; 11 –creatinine phosphate/creatine*****; 12 –O-phosphocholine; 13 – glucose; 14 – myo-inositol; 15 – creatine; 16 - UDP-glucose/UDP-N-acetyloglucosamine; 17 – NAD^+^; 18 – ATP/ADP; 19 – tyrosine; 20 – phenylalanine; 21 – AXP (ATP/AMP/ADP)*****; 22- formate; 23-AMP

****not included in the statistical and multivariate analyses***

**Tab. S3** Information about graphical representations of intracellular metabolites for each model for HEK-293 cell lines.

| **PCA -X model** | | |
| --- | --- | --- |
| **Name** | **N** | **R^2^x** |
| **Control vs 100 nM** | **6**  (3 control samples + 3 samples 100 nM) | 0,67 |
| **Control vs 0,1 mM** | **6**  (3 control samples + 3 samples 0,1 mM ) | 0,63 |
| **Control vs 3,19 mM** | **6**  (3 control samples + 3 samples 3,19 mM | 0,39 |
| **Control vs 37,8 mM** | **6**  (3 control samples + 3 samples 37,8 mM) | 0,7 |

**Tab. S4**. Information about graphical representations of intracellular metabolites for each model for COS-7 cell lines.

| **PCA -X model** | | |
| --- | --- | --- |
| **Name** | **N** | **R^2^x** |
| **Control vs 100 nM** | **6**  (3 control samples + 3 samples 100 nM) | 0,71 |
| **Control vs 1 μM** | **6**  (3 control samples + 3 samples 1 μM ) | 0,68 |
| **Control vs 0,1 mM** | **6**  (3 control samples + 3 samples 0,1 mM ) | 0,70 |
| **Control vs 3,19 mM** | **6**  (3 control samples + 3 samples 3,19 mM ) | 0,77 |
| **Control VS 37,8** **mM** | **6**  (3 control samples + 3 samples 37,8 mM ) | 0,83 |


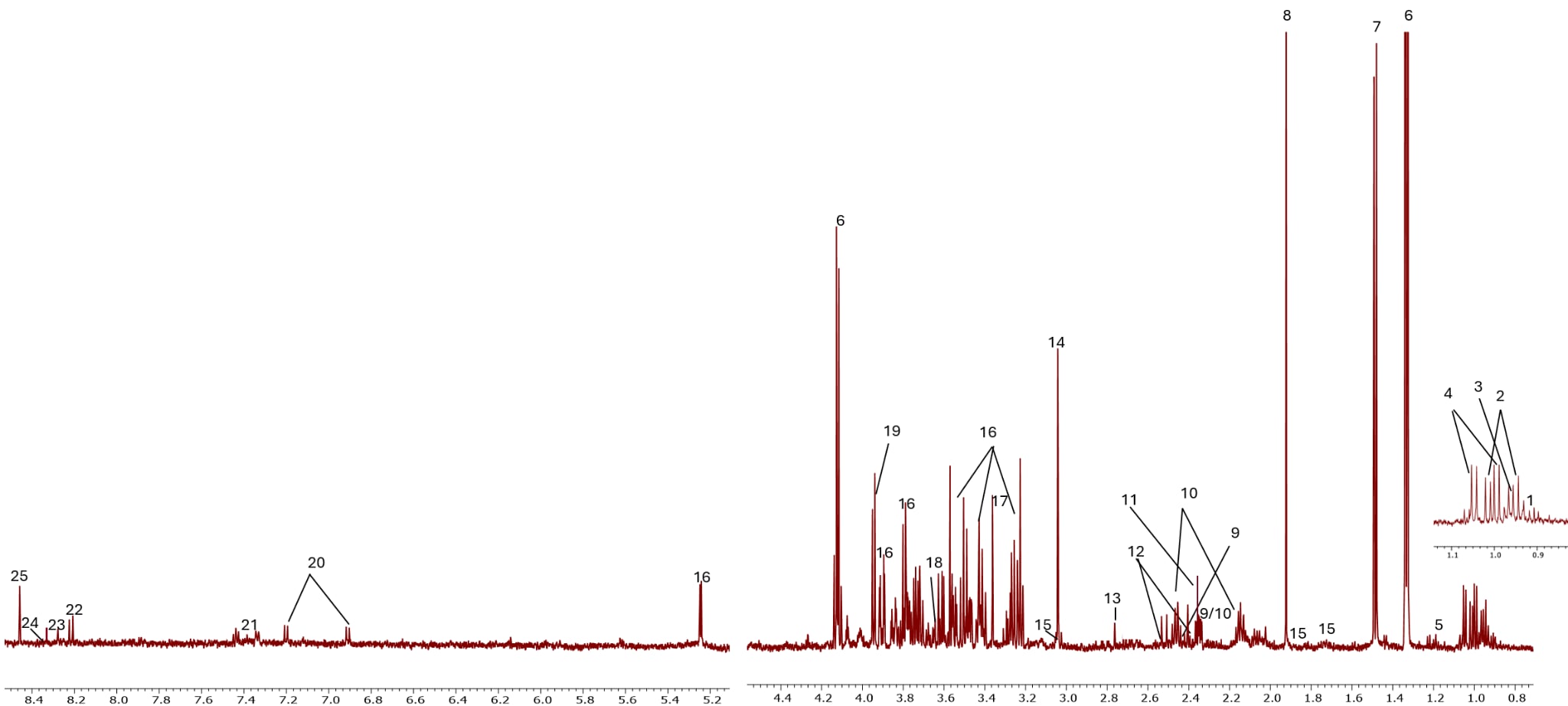


**Fig. S2 High-resolution ^1^H NMR spectrum (600 MHz) of representative spectra for HEK-293 cell line post-oxidative stress** **(3.19 mM)**. 1 – 2- hydroxyvalerate; 2 - isoleucine; 3 - leucine; 4 – valine; 5 – 3-hydroxybutyrate; 6 – lactate; 7 – alanine; 8-acetate; 9 – glutamine; 10 – glutamate; 11 – pyruvate; 12 – pyroglutamate; 13 – unknown_3; 14 – creatinine phosphate/creatine*****; 15 – lysine; 16 – glucose; 17 – methanol*****; 18 - threonine; 19 – creatine; 20 - tyrosine; 21 – phenylalanine; 22 -hypoxanthine; 23– unknown_2; 24 – unknown_1; 25 – formate

****Not included in the statistical and multivariate analyses***

**
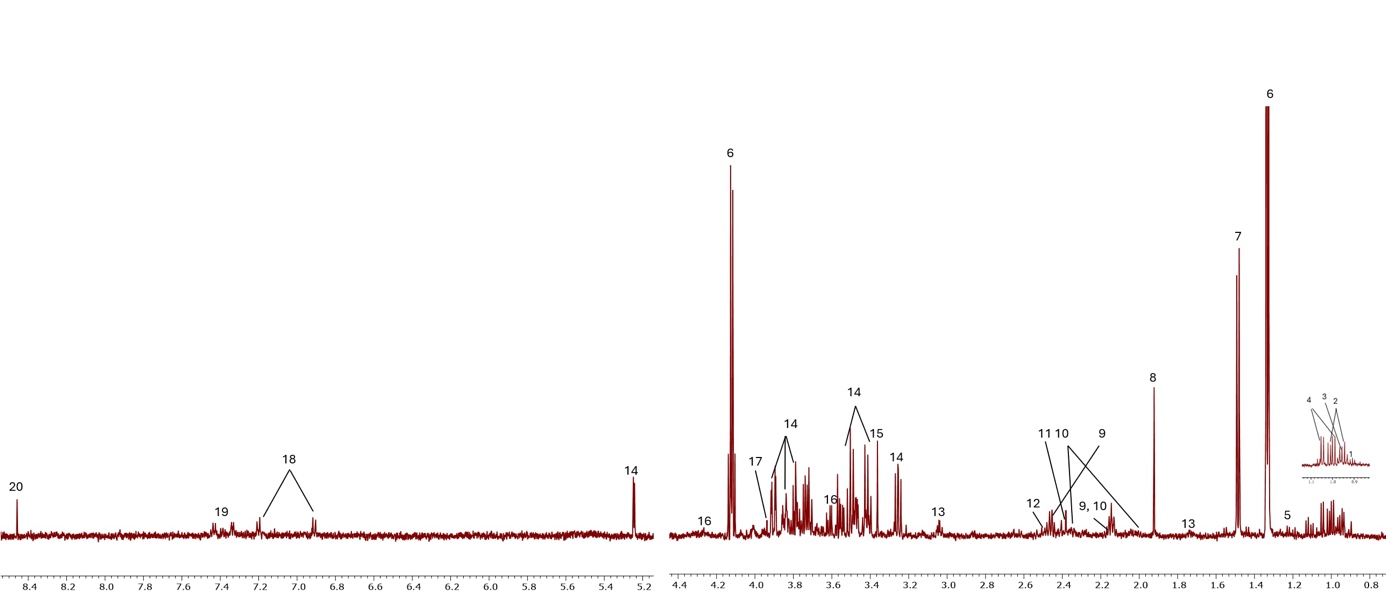
**

**Fig. S3 High-resolution ^1^H NMR spectrum (600 MHz) of representative control sample for HEK-293 cell lines (without exposure to H_2_O_2_)**

1. hydroxyvalerate; 2 - isoleucine; 3 - leucine; 4 – valine; 5 – 3-hydroxybutyrate; 6 – lactate; 7 – alanine; 8-acetate; 9 – glutamine; 10 – glutamate; 11 – pyruvate; 12 – pyroglutamate; 13 – lysine; 14 – glucose; 15 – methanol*****; 16 – threonine; 17 –creatine; 18 - tyrosine; 19 – phenylalanine; 20 – formate

****Not included in the statistical and multivariate analyses***

*
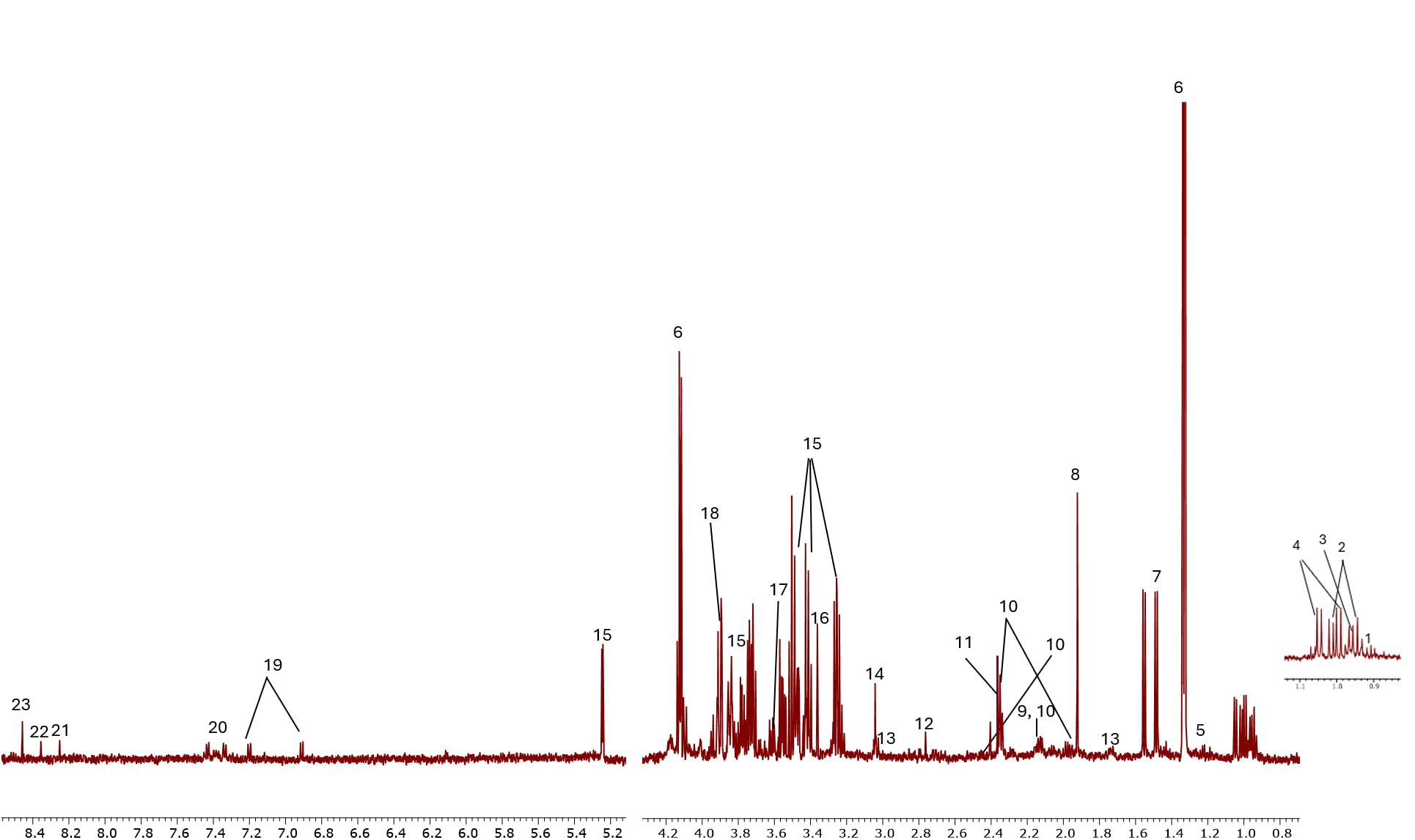
*

**Fig. S4** **High-resolution ^1^H NMR spectrum (600 MHz) of representative for COS-7 cell line post-oxidative stress (3.19 mM of H_2_O_2_).**

1 – 2- hydroxyvalerate; 2 - isoleucine; 3 - leucine; 4 – valine; 5 – 3-hydroxybutyrate; 6 – lactate; 7 – alanine; 8-acetate; 9 – glutamine; 10 – glutamate; 11 – pyruvate; 12 – unknown_3; 13- lysine; 14 – creatinine phosphate/creatine*****; 15 – glucose, 16 – methanol*****; 17 – threonine; 18 – creatine; 19 - tyrosine; 20 – phenylalanine, 21– unknown_2, 22- unknown_1; 23- formate.

****Not included in the statistical and multivariate analyses***

*
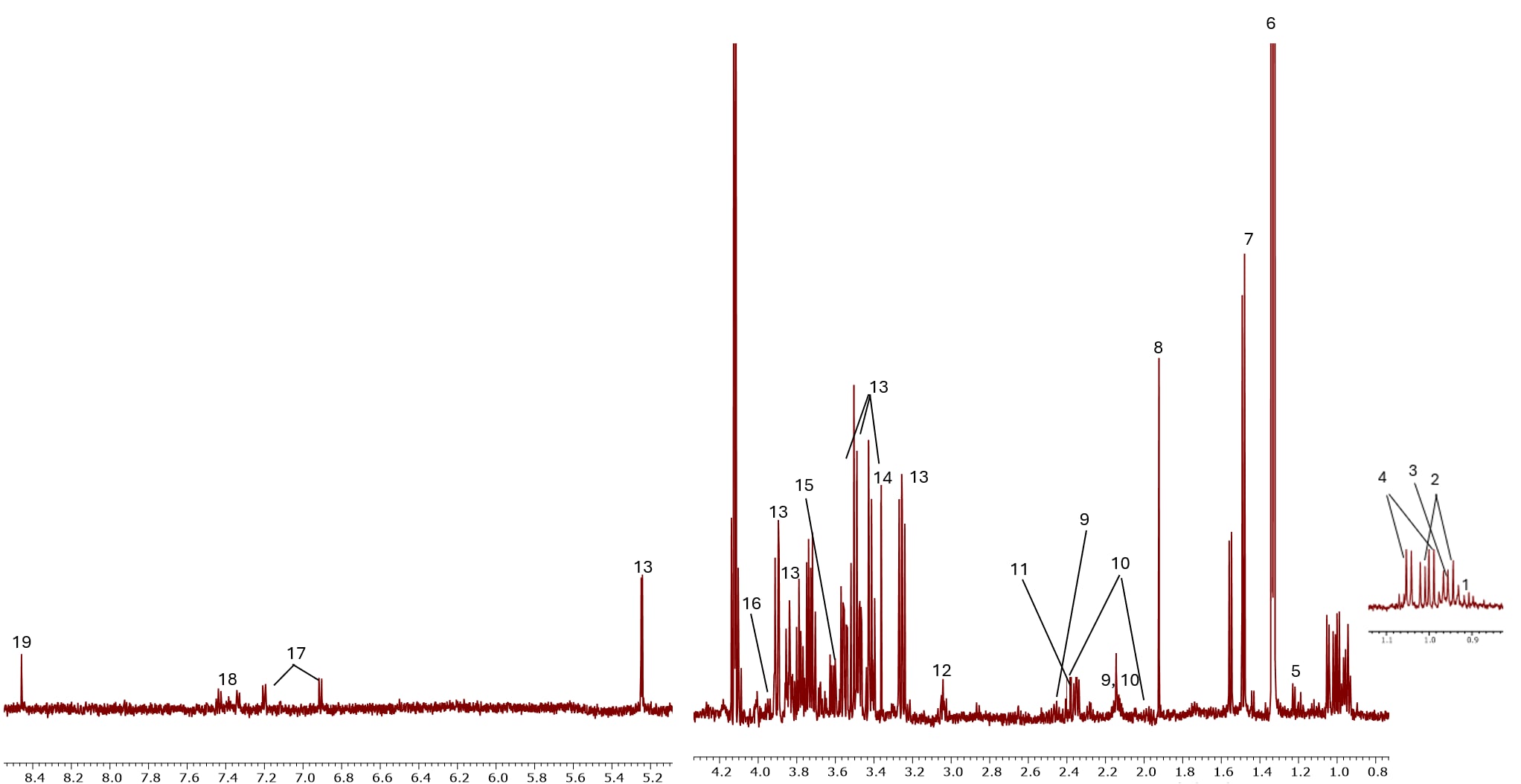
*

**Fig. S5 High resolution ^1^H NMR spectrum (600 MHz) of the representative control sample for COS-7 cell lines (without exposure to H_2_O_2_)**

1- hydroxyvalerate; 2 - isoleucine; 3 - leucine; 4 – valine; 5- 6 – 3-hydroxybutyrate; 6 – lactate; 7 – alanine; 8-acetate; 9 – glutamine; 10 – glutamate; 11 – pyruvate; 12 –lysine; 13 – glucose; 14 – methanol*****; 15– threonine; 16–creatine; 17 - tyrosine; 18 – phenylalanine; 19 – formate

****Not included in the statistical and multivariate analyses***

**Table S5**. Table comparing statistical significance and relative concentrations of metabolites for individual groups (HEK-293 cell lines, intracellular metabolites), Statistically significant metabolites are marked in orange.

| **Metabolites** | **Mean/Median** | | | | | | | | | | | | | | |
| --- | --- | --- | --- | --- | --- | --- | --- | --- | --- | --- | --- | --- | --- | --- | --- |
|  | **Control vs 100nM** | | | **Control vs 1 µM** | | | **Control vs 0,1 mM** | | | **Control vs 3,19 mM** | | | **Control vs 37,8 mM** | | |
|  | **p** | **control** | **100nM** | **p** | **control** | **1µM** | **p** | **control** | **0,1mM** | **p** | **control** | **3,19mM** | **p** | **control** | **37,8mM** |
| **NAD^+^** | 0,5928 | 0,0111 | 0,0124 | 0,2121 | 0,0111 | 0,0137 | 0,1706 | 0,0111 | 0,0098 | 0,0033 | 0,0111 | 0,0026 | 0,0041 | 0,0111 | 0,0029 |
| **ATP/ADP** | 0,1511 | 0,0844 | 0,0179 | 0,1177 | 0,0844 | 0,1055 | 0,1053 | 0,0844 | 0,071 | 0,05 | 0,0844 | 0,0616 | 0,0165 | 0,0844 | 0,02 |
| **AMP** | 0,2479 | 0,003 | 0,0018 | 0,908 | 0,003 | 0,05 | 0,2715 | 0,003 | 0,0015 | 0,0145 | 0,003 | 0,09 | 0,0381 | 0,003 | 0,0912 |
| **formate** | 0,8719 | 0,0110 | 0,0114 | 0,2519 | 0,0110 | 0,0081 | 0,705 | 0,0110 | 0,012179 | 0,1747 | 0,0110 | 0,0098 | 0,0725 | 0,0110 | 0,0779 |
| **phenylalanine** | 0,2597 | 0,0170 | 0,0169 | 0,6002 | 0,0170 | 0,0161 | 0,5326 | 0,0170 | 0,0147 | 0,2637 | 0,0170 | 0,012 | 0,0976 | 0,0170 | 0,0117 |
| **tyrosine** | 0,9976 | 0,0093 | 0,0094 | 0,5744 | 0,0093 | 0,0089 | 0,5380 | 0,0093 | 0,0081 | 0,7279 | 0,0093 | 0,0076 | 0,2516 | 0,0093 | 0,0068 |
| **UDP-glucose / UDP-N-glucosamine** | 0,9925 | 0,0586 | 0,0587 | 0,5332 | 0,0586 | 0,0632 | 0,1618 | 0,0586 | 0,0685 | 0,2091 | 0,0586 | 0,0400 | 0,0613 | 0,0586 | 0,0311 |
| **glucose** | 0,0554 | 0,0020 | 0,0007 | 0,6739 | 0,0020 | 0,0017 | 0,7567 | 0,0020 | 0,0018 | 0,07792 | 0,0020 | 0,0008 | 0,0639 | 0,0020 | 0,0009 |
| **isoleucine** | 0,5268 | 0,0103 | 0,0090 | 0,3430 | 0,0103 | 0,0084 | 0,9388 | 0,0103 | 0,0009 | 0,8974 | 0,0103 | 0,0086 | 0,5283 | 0,0103 | 0,0064 |
| **valine** | 0,9757 | 0,0311 | 0,0309 | 0,2451 | 0,0311 | 0,0262 | 0,7977 | 0,0311 | 0,0322 | 0,5886 | 0,0311 | 0,0343 | 0,5148 | 0,0311 | 0,0346 |
| **lactate** | 0,8827 | 0,1094 | 0,114 | 0,9524 | 0,1094 | 0,108 | 0,86 | 0,1094 | 0,1120 | 0,05632 | 0,1094 | 0,2071 | 0,6 | 0,1094 | 0,1078 |
| **alanine** | 0,8075 | 0,7496 | 0,7802 | 0,7146 | 0,7496 | 0,8293 | 0,3590 | 0,7496 | 0,8386 | 0,8293 | 0,7496 | 0,7816 | 0,8541 | 0,7496 | 0,7264 |
| **acetate** | 0,6606 | 0,0149 | 0,0186 | 0,8191 | 0,0149 | 0,0125 | 0,5183 | 0,0149 | 0,0208 | 0,5921 | 0,0149 | 0,0229 | 0,0598 | 0,0149 | 0,0387 |
| **creatine** | 0,4909 | 0,5217 | 0,5687 | 0,3142 | 0,5217 | 0,5890 | 0,2231 | 0,5217 | 0,4115 | 0,9570 | 0,5217 | 0,4255 | 0,05756 | 0,5217 | 0,4054 |
| **glutamate** | 0,5152 | 0,5859 | 0,6221 | 0,2436 | 0,5859 | 0,6717 | 0,0424 | 0,5859 | 0,9097 | 0,0316 | 0,5859 | 0,2339 | 0,0095 | 0,5859 | 0,3363 |
| **glutathione** | 0,5193 | 0,0716 | 0,0848 | 0,3983 | 0,0716 | 0,0890 | 0,05205 | 0,0716 | 0,0943 | 0,0506 | 0,0716 | 0,0422 | 0,0187 | 0,0716 | 0,0314 |
| **aspartate** | 0,8469 | 0,0410 | 0,0404 | 0,3871 | 0,0410 | 0,0403 | 0,5240 | 0,0410 | 0,0432 | 0,0627 | 0,0410 | 0,0308 | 0,05265 | 0,0410 | 0,029 |
| **myo-inositol** | 0,1464 | 0,1706 | 0,1982 | 0,2331 | 0,1706 | 0,2002 | 0,0668 | 0,1706 | 0,2005 | 0,9701 | 0,1706 | 0,1698 | 0,5918 | 0,1706 | 0,1902 |
| **o-phosphocholine** | 0,8074 | 0,0640 | 0,0500 | 0,9977 | 0,0640 | 0,0639 | 0,7802 | 0,0640 | 0,0490 | 0,8308 | 0,0640 | 0,0526 | 0,8044 | 0,0640 | 0,0509 |
| **leucine** | 0,8157 | 0,0128 | 0,0123 | 0,1863 | 0,0128 | 0,0105 | 0,4052 | 0,0128 | 0,0114 | 0,9837 | 0,0128 | 0,0100 | 0,5308 | 0,0128 | 0,009 |
| **pyruvate** | 0,9 | 0,009 | 0,0092 | 0,59 | 0,009 | 0,007 | 0,45 | 0,009 | 0,0072 | 0,0094 | 0,009 | 0,0043 | 0,007 | 0,009 | 0,0017 |

**Table S6**. Table comparing statistical significance and relative concentrations of metabolites for individual groups (COS-7 cell lines, intracellular metabolites), Statistically significant metabolites are marked in orange.

| **Metabolites** | **Mean/Median** | | | | | | | | | | | | | | | | |
| --- | --- | --- | --- | --- | --- | --- | --- | --- | --- | --- | --- | --- | --- | --- | --- | --- | --- |
|  | **Control vs 100nM** | | | **Control vs 1 µM** | | | | **Control vs 0,1 mM** | | | **Control vs 3,19 mM** | | | | **Control vs 37,8 mM** | | |
|  | **p** | **control** | **100nM** | **p** | **control** | **1µM** | **p** | | **control** | **0,1mM** | **p** | **control** | **3,19mM** | **p** | | **control** | **37,8mM** |
| **NAD+** | 0,5533 | 0,0225 | 0,0196 | 0,0870 | 0,0225 | 0,0163 | 0,4143 | | 0,0225 | 0,0190 | 0,0077 | 0,0225 | 0,0077 | 0,0269 | | 0,0225 | 0,0085 |
| **ATP /ADP** | 0,8480 | 0,0823 | 0,0809 | 0,2992 | 0,0823 | 0,0742 | 0,6453 | | 0,0823 | 0,0778 | 0,0514 | 0,0823 | 0,0573 | 0,009 | | 0,0823 | 0,0281 |
| **AMP** | 0,6839 | 0,0051 | 0,004 | 0,4139 | 0,0051 | 0,00351 | 0,5832 | | 0,0051 | 0,0039 | 0,0140 | 0,0051 | 0,014 | 0,008 | | 0,0051 | 0,024 |
| **formate** | 0,7293 | 0,0085 | 0,0090 | 0,5742 | 0,0085 | 0,0074 | 0,2190 | | 0,0085 | 0,0086 | 0,06014 | 0,0085 | 0,00917 | 0,0091 | | 0,0085 | 0,0206 |
| **phenylalanine** | 0,3588 | 0,0166 | 0,0151 | 0,7614 | 0,0166 | 0,0158 | 0,3026 | | 0,0166 | 0,0129 | 0,4796 | 0,0166 | 0,0202 | 0,2414 | | 0,0166 | 0,0215 |

| **tyrosine** | 0,8472 | 0,0176 | 0,0169 | 0,2364 | 0,0176 | 0,0142 | 0,396 | 0,0176 | 0,0169 | 0,4987 | 0,0176 | 0,0201 | 0,3814 | 0,0176 | 0,0213 |
| --- | --- | --- | --- | --- | --- | --- | --- | --- | --- | --- | --- | --- | --- | --- | --- |
| **UDP-glucose / UDP-N-glucosamine** | 0,8949 | 0,0851 | 0,0883 | 0,7590 | 0,0851 | 0,0781 | 0,6106 | 0,0851 | 0,0722 | 0,3469 | 0,0851 | 0,0608 | 0,3589 | 0,0851 | 0,0621 |
| **glucose** | 0,7993 | 0,0253 | 0,0233 | 0,9567 | 0,0253 | 0,0249 | 0,8391 | 0,0253 | 0,0265 | 0,6388 | 0,0253 | 0,0286 | 0,7936 | 0,0253 | 0,0175 |
| **isoleucine** | 0,6910 | 0,0130 | 0,0148 | 0,8382 | 0,0130 | 0,0119 | 0,6622 | 0,0130 | 0,0142 | 0,1626 | 0,130 | 0,0169 | 0,045 | 0,0130 | 0,0221 |
| **valine** | 0,6235 | 0,0331 | 0,0382 | 0,7236 | 0,0331 | 0,0292 | 0,9167 | 0,0331 | 0,0340 | 0,1082 | 0,0331 | 0,0512 | 0,0337 | 0,0331 | 0,0574 |
| **lactate** | 0,8120 | 0,564 | 0,574 | 0,9525 | 0,564 | 0,583 | 0,8684 | 0,564 | 0,6742 | 0,61 | 0564 | 0,568 | 0,56 | 0,567 | 0,513 |
| **alanine** | 0,8958 | 0,5286 | 0,5341 | 0,2433 | 0,5286 | 0,3047 | 0,2651 | 0,5286 | 0,3174 | 0,1791 | 0,5286 | 0,2869 | 0,1175 | 0,5286 | 0,3663 |
| **acetate** | 0,8942 | 10,4709 | 10,3458 | 0,1431 | 10,4709 | 9,3228 | 0,3276 | 10,4709 | 9,7688 | 0,1322 | 10,4709 | 9,1774 | 0,0599 | 10,4709 | 8,7142 |
| **creatine** | 0,6688 | 0,3700 | 0,3960 | 0,6058 | 0,3700 | 0,3459 | 0,9898 | 0,3700 | 0,3709 | 0,3744 | 0,3700 | 0,3020 | 0,0061 | 0,3700 | 0,2087 |
| **glutamate** | 0,9469 | 0,4409 | 0,4449 | 0,5681 | 0,4409 | 0,4096 | 0,8232 | 0,4409 | 0,4248 | 0,0067 | 0,4409 | 0,1810 | 0,0027 | 0,4409 | 0,1182 |
| **aspartate** | 0,9906 | 0,0747 | 0,0748 | 0,1394 | 0,0747 | 0,0561 | 0,1517 | 0,0747 | 0,0603 | 0,0504 | 0,0747 | 0,0377 | 0,009 | 0,0747 | 0,0111 |
| **myo-inositol** | 0,7764 | 0,2661 | 0,2436 | 0,6507 | 0,2661 | 0,2297 | 0,8242 | 0,2661 | 0,2462 | 0,9810 | 0,2661 | 0,2639 | 0,7532 | 0,2661 | 0,2927 |
| **o-phosphocholine** | 0,8761 | 0,2217 | 0,2131 | 0,7575 | 0,2217 | 0,2109 | 0,9118 | 0,2217 | 0,2274 | 0,8671 | 0,2217 | 0,2305 | 0,8638 | 0,2217 | 0,2325 |
| **glutathione** | 0,6439 | 0,00812 | 0,1377 | 0,4600 | 0,00812 | 0,016 | 0,057 | 0,00812 | 0,0085 | 0,0189 | 0,00812 | 0,00415 | 0,0026 | 0,00812 | 0,00298 |
| **pyruvate** | 0,923 | 0,0137 | 0,0136 | 0,8976 | 0,0137 | 0,0139 | 0,07 | 0,0137 | 0,01378 | 0,051 | 0,0137 | 0,0138 | 0,004 | 0,0137 | 0,0045 |
| **leucine** | 0,9345 | 0,009 | 0,0113 | 0,7 | 0,009 | 0,0132 | 0,656 | 0,009 | 0,0104 | 0,9837 | 0,009 | 0,088 | 0,5308 | 0,009 | 0,007 |

**Tab. S7** Information about graphical representations for each model for HEK-293 cell lines.

| **PCA -X model** | | |
| --- | --- | --- |
| **Name** | **N** | **R^2^x** |
| **Control vs 100 nM** | **12**  (6 control samples + 6 samples 100 nM) | 0,59 |
| **Control vs 1 μM** | **12**  (6 control samples + 6 samples 1 μM ) | 0,73 |
| **Control vs 0,1 mM** | **12**  (6 control samples + 5 samples 0,1 mM ) | 0,63 |
| **Control vs 3,19 mM** | **11**  (6 control samples + 5 samples 3,19 mM ) | 0,83 |
| **Control VS 37,8** **mM** | **11**  (6 control samples + 5 samples 37,8 mM ) | 0,91 |

**Tab. S8** Information about graphical representations for each model for COS-7 cell lines.

| **PCA -X model** | | |
| --- | --- | --- |
| **Name** | **N** | **R^2^x** |
| **Control vs 100 nM** | **12**  (6 control samples + 6 samples 100 nM) | 0,45 |
| **Control vs 1 μM** | **12**  (6 control samples + 6 samples 1 μM ) | 0,51 |
| **Control vs 0,1mM** | **12**  (6 control samples + 6 samples 0,1 mM ) | 0,56 |
| **Control vs 3,19 mM** | **12**  (6 control samples + 6 samples 3,19 mM ) | 0,55 |
| **Control VS 37,8** **mM** | **11**  (6 control samples + 5 samples 37,8 mM ) | 0,88 |

**Table S9**. Table comparing statistical significance and relative concentrations of metabolites for individual groups (HEK-293 cell lines, extracellular metabolites), Statistically significant metabolites are marked in orange.

| **Metabolites** | **Mean/Median** | | | | | | | | | | | | | | |
| --- | --- | --- | --- | --- | --- | --- | --- | --- | --- | --- | --- | --- | --- | --- | --- |
|  | **Control vs 100nM** | | | **Control vs 1 µM** | | | **Control vs 0,1 mM** | | | **Control vs 3,19 mM** | | | **Control vs 37,8 mM** | | |
|  | **p** | **control** | **100nM** | **p** | **control** | **1µM** | **p** | **control** | **0,1mM** | **p** | **control** | **3,19mM** | **p** | **control** | **37,8mM** |
| **leucine** | 0,240 | 0,0146 | 0,001 | 0,240 | 0,0146 | 0,0146 | 0,177 | 0,0146 | 0,015 | 0,8598 | 0,0146 | 0,0143 | 0,3819 | 0,0146 | 0,0132 |
| **isoleucine** | 0,879 | 0,0119 | 0,0120 | 0,592 | 0,0119 | 0,0112 | 0,9324 | 0,0119 | 0,0120 | 0,83 | 0,0119 | 0,01167 | 0,9307 | 0,0119 | 0,01 |
| **valine** | 0,94 | 0,03 | 0,03 | 0,394 | 0,03 | 0,031 | 0,09 | 0,030 | 0,026 | 0,0020 | 0,03 | 0,035 | 0,09 | 0,030 | 0,027 |
| **2-hydroxyvalerate** | 0,94 | 0,001 | 0,0012 | 0,394 | 0,001 | 0,002 | 0,9 | 0,001 | 0,0014 | 0,13 | 0,001 | 0,00152 | 0,100 | 0,001 | 0,0157 |
| **acetate** | 0,567 | 0,05 | 0,052 | 0,837 | 0,05 | 0,0045 | 0,017 | 0,05 | 0,08 | 0,0043 | 0,05 | 0,14 | 0,0043 | 0,05 | 0,13 |
| **glutamine** | 0,24 | 0,03 | 0,03 | 0,593 | 0,03 | 0,025 | 0,04 | 0,03 | 0,018 | 0,0033 | 0,03 | 0,0018 | 0,0037 | 0,03 | 0,004 |
| **glutamate** | 0,671 | 0,0230 | 0,0287 | 0,700 | 0,0230 | 0,0183 | 0,0463 | 0,0230 | 0,037 | 0,0420 | 0,0230 | 0,038 | 0,0080 | 0,0230 | 0,047 |
| **pyruvate** | 0,437 | 0,0187 | 0,02 | 1 | 0,0187 | 0,025 | 0,9307 | 0,0187 | 0,01 | 0,051 | 0,0187 | 0,037 | 0,0395 | 0,0187 | 0,127 |
| **glucose** | 0,31 | 0,0509 | 0,07 | 0,589 | 0,0509 | 0,08 | 0,3290 | 0,0509 | 0,0423 | 0,3290 | 0,0509 | 0,04096 | 0,0602 | 0,0509 | 0,0356 |
| **lactate** | 0,937 | 0,005 | 0,0045 | 0,699 | 0,005 | 0,0057 | 0,05 | 0,005 | 0,003 | 0,048 | 0,005 | 0,0023 | 0,004 | 0,005 | 0,012 |
| **unknown_2** | 0,377 | 0,001 | 0,001 | 0,160 | 0,001 | 0,0012 | 0,0663 | 0,001 | 0,009 | 0,0085 | 0,001 | 0,01577 | 0,0111 | 0,001 | 0,0371 |
| **alanine** | 0,605 | 0,21 | 0,221 | 0,712 | 0,21 | 0,24 | 0,668 | 0,21 | 0,214 | 0,9712 | 0,21 | 0,208 | 0,218 | 0,21 | 0,177 |
| **formate** | 0,918 | 0,0006 | 0,00065 | 0,077 | 0,0006 | 0,00056 | 0,79 | 0,0006 | 0,00072 | 0,0085 | 0,0006 | 0,0031 | 0,0496 | 0,0006 | 0,003 |
| **phenylalanine** | 0,917 | 0,002 | 0,002 | 0,800 | 0,002 | 0,001 | 0,7259 | 0,002 | 0,0018 | 0,7872 | 0,002 | 0,0127 | 0,1372 | 0,002 | 0,0142 |
| **tyrosine** | 0,919 | 0,0146 | 0,015 | 0,16 | 0,0146 | 0,0147 | 0,7577 | 0,0146 | 0,0155 | 0,7872 | 0,0146 | 0,0143 | 0,1232 | 0,0146 | 0,0132 |
| **pyroglutamate** | 0,886 | 0,0009 | 0,00099 | 0,294 | 0,0009 | 0,000131 | 0,02 | 0,0009 | 0,0017 | 0,002 | 0,0009 | 0,002506 | 0,01 | 0,0009 | 0,002251 |
| **unknown_3** | 0,494 | 0,0014 | 0,001 | 0,322 | 0,0014 | 0,0015 | 0,0775 | 0,0014 | 0,002 | 0,0005 | 0,0014 | 0,0045 | 0,001 | 0,0014 | 0,005 |
| **3-hydroxybutyrate** | 0,4 | 0,015 | 0,012 | 0,322 | 0,015 | 0,02 | 0,028 | 0,015 | 0,22 | 0,003 | 0,015 | 0,346 | 0,009 | 0,015 | 0,387 |
| **threonine** | 0,879 | 0,0319 | 0,0320 | 0,592 | 0,0319 | 0,0312 | 0,9324 | 0,0319 | 0,0320 | 0,1255 | 0,0319 | 0,0327 | 0,9307 | 0,0319 | 0,0317 |
| **lysine** | 0,9 | 0,0210 | 0,0225 | 0,592 | 0,0210 | 0,0220 | 0,9254 | 0,0210 | 0,0220 | 0,3456 | 0,0210 | 0,0100 | 0,897 | 0,0210 | 0,0120 |
| **Hypoxanthine** | 0,903 | 0,0015 | 0,0015 | 0,171 | 0,0015 | 0,0018 | 0,1255 | 0,0015 | 0,0018 | 0,011 | 0,0015 | 0,0021 | 0,002 | 0,0015 | 0,0046 |
| **Unknown_1** | 0, 123 | 0,0002 | 0,00021 | 0,228 | 0,0002 | 0,00023 | 0,049 | 0,0002 | 0,0033 | 0,012 | 0,0002 | 0,005 | 0,009 | 0,0002 | 0,0054 |

**Table S10** Table comparing statistical significance and relative concentrations of metabolites for individual groups (COS-7 cell lines, extracellular metabolites), Statistically significant metabolites are marked in orange.

| **Metabolites** | **Mean/Median** | | | | | | | | | | | | | | | |
| --- | --- | --- | --- | --- | --- | --- | --- | --- | --- | --- | --- | --- | --- | --- | --- | --- |
|  | **Control vs 100nM** | | | **Control vs 1 µM** | | | **Control vs 0,1 mM** | | | | **Control vs 3,19 mM** | | | **Control vs 37,8 mM** | | |
|  | **p** | **control** | **100nM** | **p** | **control** | **1µM** | **p** | **control** | **0,1mM** | **p** | | **control** | **3,19mM** | **p** | **control** | **37,8mM** |
| **formate** | 0,6857 | 0,0061 | 0,0067 | 0,7778 | 0,0061 | 0,0058 | 0,9127 | 0,0061 | 0,0062 | 0,2333 | | 0,0061 | 0,0075 | 0,2548 | 0,0061 | 0,0074 |
| **tyrosine** | 0,1320 | 0,0016 | 0,0056 | 0,6991 | 0,0016 | 0,0192 | 0,3939 | 0,0016 | 0,0040 | 0,2403 | | 0,0016 | 0,0028 | 0,2403 | 0,0016 | 0,0026 |
| **unknown_1** | 0,2560 | 0,001 | 0,0019 | 0,6685 | 0,001 | 0,0012 | 0,048 | 0,001 | 0,0017 | 0,0260 | | 0,001 | 0,0032 | 0,0317 | 0,001 | 0,0036 |
| **unknown_2** | 0,8182 | 0,00015 | 0,00016 | 0,8182 | 0,00015 | 0,000152 | 0,1797 | 0,00015 | 0,00024 | 0,0031 | | 0,00015 | 0,0031 | 0,0049 | 0,00015 | 0,0040 |
| **phenylalanine** | 0,1731 | 0,0121 | 0,0227 | 0,6932 | 0,0121 | 0,0134 | 0,4288 | 0,0121 | 0,0140 | 0,5191 | | 0,0121 | 0,0136 | 0,7675 | 0,0121 | 0,0114 |
| **threonine** | 0,999 | 0,0312 | 0,0320 | 0,734 | 0,0312 | 0,0312 | 0,9324 | 0,0312 | 0,023 | 0,236 | | 0,0312 | 0,01 | 0,07 | 0,0312 | 0,005 |
| **isoleucine** | 0,6177 | 0,0295 | 0,0278 | 0,0677 | 0,0295 | 0,0237 | 0,0109 | 0,0295 | 0,0204 | 0,0504 | | 0,0295 | 0,0231 | 0,0501 | 0,0295 | 0,0218 |
| **leucine** | 0,3095 | 0,0289 | 0,0287 | 0,5747 | 0,0289 | 0,0276 | 0,0961 | 0,0289 | 0,0245 | 0,3287 | | 0,0289 | 0,0265 | 0,1403 | 0,0289 | 0,0240 |
| **valine** | 0,3939 | 0,0315 | 0,0310 | 0,9372 | 0,0315 | 0,0292 | 1,0000 | 0,0315 | 0,0282 | 1,0000 | | 0,0315 | 0,0288 | 0,1797 | 0,0315 | 0,0253 |
| **2-hydroxyvalerate** | 0,3636 | 0,0026 | 0,0019 | 0,1527 | 0,0026 | 0,0020 | 0,077 | 0,0026 | 0,0023 | 0,2358 | | 0,0026 | 0,0019 | 0,2008 | 0,0026 | 0,0020 |
| **lactate** | 0,5134 | 1,1500 | 1,3804 | 0,5145 | 1,1500 | 1,0584 | 0,5579 | 1,1500 | 1,0564 | 0,3912 | | 1,1500 | 1,0764 | 0,02733 | 1,1500 | 0,931 |
| **acetate** | 0,8182 | 0,0322 | 0,0297 | 1,0000 | 0,0322 | 0,0305 | 0,1797 | 0,0322 | 0,0407 | 0,0411 | | 0,0322 | 0,0493 | 0,0394 | 0,0322 | 0,0497 |
| **glutamate** | 0,2662 | 0,0637 | 0,0731 | 0,7737 | 0,0637 | 0,0615 | 0,8326 | 0,0637 | 0,0615 | 0,0501 | | 0,0637 | 0,07 | 0,017 | 0,0637 | 0,080 |
| **pyruvate** | 0,4848 | 0,0090 | 0,0153 | 0,1543 | 0,0090 | 0,0084 | 0, 140 | 0,0090 | 0,007 | 0,0092 | | 0,0090 | 0,006 | 0,0132 | 0,0090 | 0,0125 |
| **glutamine** | 0,3354 | 0,0429 | 0,0486 | 0,7455 | 0,0429 | 0,0413 | 0,9812 | 0,0429 | 0,0428 | 0,02555 | | 0,0429 | 0,01478 | 0,024848 | 0,0429 | 0,00936 |
| **3-hydroxybutyrate** | 0,6 | 0,02 | 0,023 | 0,322 | 0,02 | 0,024 | 0,2 | 0,02 | 0,028 | 0,1 | | 0,02 | 0,0313 | 0,055 | 0,019 | 0,0382 |
| **glucose** | 0,3186 | 0,1274 | 0,1551 | 0,9098 | 0,1274 | 0,1295 | 0,9983 | 0,1274 | 0,1274 | 0,3648 | | 0,1274 | 0,1410 | 0,6150 | 0,1274 | 0,1353 |
| **unknown_3** | 0,494 | 0,003 | 0,003 | 0,322 | 0,003 | 0,004 | 0,00175 | 0,003 | 0,0055 | 0,0005 | | 0,003 | 0,00566 | 0,0003 | 0,003 | 0,008 |
| **lysine** | 0,961 | 0,0315 | 0,0325 | 0,763 | 0,0315 | 0,0300 | 0,986 | 0,0315 | 0,0320 | 0,7456 | | 0,0315 | 0,0300 | 0,677 | 0,0315 | 0,0320 |
| **alanine** | 0,705 | 0,02 | 0,003 | 0,712 | 0,02 | 0,004 | 0,899 | 0,02 | 0,0050 | 0,998 | | 0,02 | 0,011 | 0,6302 | 0,02 | 0,019 |
